# Modeling human retinoblastoma using embryonic stem cell-derived retinal organoids

**DOI:** 10.1016/j.xpro.2021.100444

**Published:** 2021-04-07

**Authors:** Hui Liu, Zi-Qi Hua, Zi-Bing Jin

**Affiliations:** 1 Laboratory of Stem Cell & Retinal Regeneration, The Eye Hospital, Wenzhou Medical University, Wenzhou 325027, China; 2 Institute of Biomedical Big Data, School of Biomedical Engineering, School of Ophthalmology & Optometry and Eye Hospital, Wenzhou Medical University, Wenzhou 325027, China; 3 Beijing Institute of Ophthalmology, Beijing Tongren Eye Center, Beijing Tongren Hospital, Capital Medical University, Beijing Ophthalmology & Visual Sciences Key Laboratory, Beijing 100730, China; 4 Institute of Stem Cell Research, The Eye Hospital, Wenzhou Medical University, Wenzhou 325027, China

**Keywords:** Cell culture, Cancer, Stem Cells, Cell Differentiation, Organoids

## Abstract

Retinoblastoma (Rb) is the most prevalent intraocular malignancy in early childhood. Traditional models are unable to accurately recapitulate the origin and development of human Rb. Here, we present a protocol to establish a novel human Rb organoid (hRBO) model derived from genetically engineered human embryonic stem cells (hESCs). This hRBO model exhibits properties highly consistent with human primary Rb and can be used effectively for dissecting the origination and pathogenesis of Rb as well as for screening of potential therapies.

For complete details on the use and execution of this protocol, please refer to Liu et al. (2020).

## Highlights

• Detailed protocol to generate hESCs with mutagenesis of the RB1 gene
• Protocol to generate an in-dish human Rb organoid system from RB1 mutant hESCs
• Steps for quality control and assessment of hRBO
• Human Rb organoids (hRBO) recapitulate key features of human primary Rb

## Graphical abstract

**Figure:**
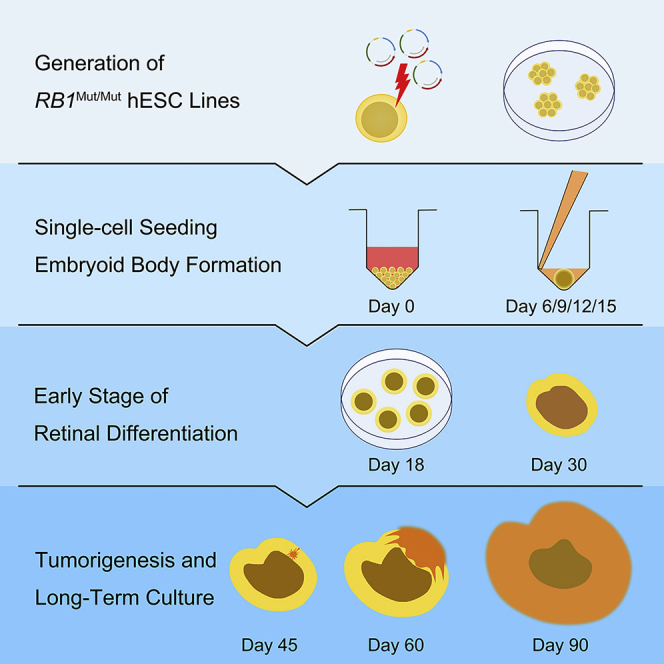

## Before you begin

Prepare the below materials before starting this protocol. Refer to key resources table for the complete list of required materials and equipment.

***Note:*** All laboratory procedures are performed in a Class II biological hood under sterile conditions.

### Cell lines

1. Undifferentiated H9 hESCs are obtained from the WiCell Research Institute, which are genetically engineered and used to generate human Rb organoids (hRBOs) in this protocol.

***Alternatives:*** In our experience, this protocol can be performed with other hESC lines.

***Note:*** Research using hESCs must be conducted in accordance with the respective legal and ethical guidelines. hESCs and their derivatives should be regularly checked to ensure that they are not infected with mycoplasma.

### Targeting strategy and guide RNA design

**Timing: 2 weeks**

2. The nonsense mutation c.958C>T (p.R320X) of *RB1* gene is the most frequent mutation associated with predisposition to Rb (http://rb1-lovd.d-lohmann.de). Thus, we generated a p.R320X knockin mutation in hESCs using CRISPR/Cas9 genome-editing technology.
3. Analyze the structure of the *RB1* gene, and then design targeting strategy (Figure 1A).

**Figure 1:**
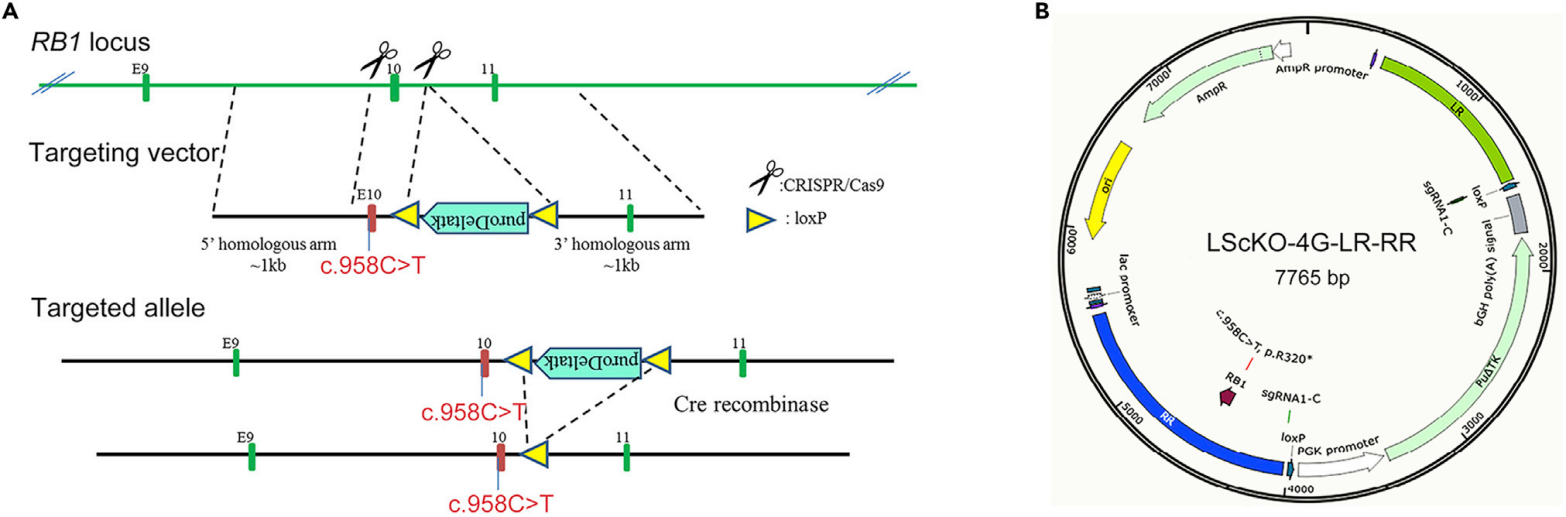
CRISPR/Cas9-mediated genome editing (A) Targeting strategy for the generation of *RB1*^Mut/Mut^ hESCs including mutation knockin and resistance gene remove. (B) Sequence map for LScKO-4G-*RB1* targeting vector (LScKO-4G-LR-RR).
4. Use the CRISPR Design Tool (https://zlab.bio/guide-design-resources) to design guide RNAs (gRNAs) on the *RB1* target sequence, which can be obtained from USCS Genome Browser, human genome version GRCh38/hg38.
5. Open the CRISPR Design Tool web, and paste the target sequence in the ‘sequence’ box and select ‘unique genomic region’. Enter your e-mail address and the gene name into the appropriate fields. Click ‘Submit Query’. Guide sequences will be displayed as they are searched, including specificity scores and off-target hits.
6. Choose the sequences of 10–15 of the top-scoring gRNAs, and evaluate their CRISPR/Cas9 genome editing activity using Universal Crispr RNA activity assay (Biocytogen) according to manufacturer’s instruction, and select appropriate gRNAs by considering factors including activity and specificity.

***Alternatives:*** Other kits or methods can also be used to estimate the genome editing activity for candidate gRNAs.

### pCS-sgRNA plasmid and CRISPR/Cas9 targeting vector construction

**Timing: 2–3 weeks**

7. A valid gRNA (gRNA 1 sequence: 5′-AGGAAAGTTGCTTGAACCCCGGG-3′) is selected and cloned into pCS-3G vector (Biocytogen) to generate the CRISPR/Cas9 guide-carrying plasmid pCS-3G-gRNA1.
8. The LScKO-4G-*RB1* targeting vector (LScKO-4G-LR-RR) corresponding to the human *RB1* gene containing c.958C>T; p.R320X mutation was designed and constructed by Biocytogen (Figure 1B).

## Key resources table

**Table:** 

REAGENT or RESOURCE	SOURCE	IDENTIFIER
**Antibodies**		

Anti-Ki67 antibody	Abcam	Cat# ab15580; RRID:AB_443209
Anti-Ki-67 Clone B56 (RUO)	BD Biosciences	Cat# 556003; RRID:AB_396287
Alexa Fluor 488 Mouse anti-Oct3/4 Clone 40/Oct-3	BD Biosciences	Cat# 560253; RRID:AB_1645304
Alexa Fluor 647 Mouse anti-Ki-67 Clone B56	BD Biosciences	Cat# 558615; RRID:AB_647130
Purified Mouse Anti-Human DEK Clone 2/DEK	BD Biosciences	Cat# 610948; RRID:AB_398261
Anti-CDKN2A/p16INK4a	Abcam	Cat# ab108349; RRID:AB_10858268
Anti-Syk (D3Z1E)	Cell Signaling Technology	Cat# 13198; RRID:AB_2687924
Anti-Syk (4D10)	Santa Cruz Biotechnology	Cat# sc-1240; RRID:AB_628308
Anti-NSE antibody [EPR3377]	Abcam	Cat# ab79757; RRID:AB_1603753
Anti-Rb antibody [EPR17512]	Abcam	Cat# ab181616
Anti-p53 (DO-1)	Santa Cruz Biotechnology	Cat# sc-126; RRID:AB_628082
Anti-GAPDH	Abcam	Cat# ab181602; RRID:AB_2630358
DAPI	Thermo Fisher Scientific	Cat# D1306; RRID:AB_2629482
Donkey anti-Goat IgG (H+L) Secondary Antibody, Alexa Fluor Plus 488	Thermo Fisher Scientific	Cat# A32814; RRID:AB_2762838
Goat anti-Mouse IgG (H+L) Secondary Antibody, Alexa Fluor Plus 488	Thermo Fisher Scientific	Cat# A32723; RRID:AB_2633275
Alexa Fluor 594 AffiniPure Donkey Anti-Mouse IgG (H+L)	Jackson ImmunoResearch	Cat# 715-585-151; RRID:AB_2340855
Donkey anti-Rabbit IgG (H+L) Secondary Antibody, Alexa Fluor 594	Thermo Fisher Scientific	Cat# A-21207; RRID:AB_141637
Donkey anti-Goat IgG (H+L) Secondary Antibody, Alexa Fluor 555 conjugate	Thermo Fisher Scientific	Cat# A-21432; RRID:AB_2535853
IRDye 800CW Goat anti-Rabbit IgG Secondary Antibody	LI-COR	Cat# 926-32211; RRID:AB_621843

**Chemicals, peptides, and recombinant proteins**		

EDTA (0.5 M), pH 8.0, RNase-free	Thermo Fisher Scientific	Cat# AM9261
ACCUTASE	Stem Cell Technologies	Cat# 07920
Certified Fetal Bovine Serum, Qualified for Human Embryonic Stem Cells	Biological Industries	Cat# 04-002-1A
Growth Factor Reduced (GFR) Basement Membrane Matrix (Matrigel)	Corning	Cat# 356231
DMEM/F-12, GlutaMAX	Thermo Fisher Scientific	Cat# 10565042
DNase I	Roche	Cat# 11284932001
GlutaMAX Supplement	Thermo Fisher Scientific	Cat# 35050061
Ham's F-12 Nutrient Mix (Hams F12)	Thermo Fisher Scientific	Cat# 11765054
Iscove’s Modified Dulbecco’s Medium (IMDM)	Thermo Fisher Scientific	Cat# 12440053
KnockOut Serum Replacement (KSR)	Thermo Fisher Scientific	Cat# 10828028
mTeSR-1 media	Stem Cell Technologies	Cat# 85850
N-2 Supplement (100×)	Thermo Fisher Scientific	Cat# 17502048
NEG-50 Frozen Section Medium	Thermo Fisher Scientific	Cat# 6502
Penicillin-Streptomycin (PS)	Thermo Fisher Scientific	Cat# 15140163
Paraformaldehyde (PFA)	Beyotime Biotechnology	Cat# P0098
Puromycin	Gene Operation	Cat# ISY1130-0025MG
Recombinant Human BMP-4 Protein (hBMP4)	R&D Systems	Cat# 314-BP
Retinoic Acid	Sigma-Aldrich	Cat# R2625
Taurine	Sigma-Aldrich	Cat# T8691
TeSR-E8	Stem Cell Technologies	Cat# 5990
BSA, Fraction V	Beyotime Biotechnology	Cat# ST023
Triton X-100	Sangon Biotech	Cat# A600198-0500
TrypLE Select (1×)	Thermo Fisher Scientific	Cat# 12563011
Y-27632 2HCl	Selleck	Cat# S1049
KaryoMAX Colcemid Solution	Thermo Fisher Scientific	Cat# 15212
Propidium iodide	Sigma-Aldrich	Cat# P4170
RNase A solution	Sigma-Aldrich	Cat# R6148
Pentobarbital sodium salt	Sigma-Aldrich	Cat# P3761
Protease Inhibitor Cocktail	Thermo Fisher Scientific	Cat# 87785

**Critical commercial assays**		

DNeasy Tissue Kit	SimGEN	Cat# 3101050
DNeasy Blood & Tissue Kit	QIAGEN	Cat# 69504
RNeasy Mini Kit (50)	QIAGEN	Cat# 74104
RNA Nano 6000 Assay Kit	Agilent Technologies	Cat# 5067-1511
P3 Primary Cell 4D-Nucleofector X Kit S	Lonza	Cat# V4XP-3032

**Experimental models: cell lines**		

H9 hESCs	WiCell	Thomson et al., 1998
*RB1*^Mut/Mut^ hESCs (c.958C>T)	This paper	N/A
*RB1*^−/−^ hESCs (targeting exon 1 of RB1)	This paper	N/A

**Oligonucleotides**		

*RB1* mutation knockin sgRNAs	This paper	N/A
*RB1* knockout sgRNA: CACCGCGGTGGCGGCCGTTTTTCGG	This paper	N/A

**Recombinant DNA**		

*RB1* mutation knockin guide-carrying plasmid: pCS-3G	Biocytogen	N/A
*RB1* mutation knockin targeting vector: LScKO-4G-LR-RR	Biocytogen	N/A
*RB1* knockout guide-carrying plasmid: pX330-U6-Chimeric BB-CBh-hSpCas9-2A-Puro	Addgene	Cat# 42230

**Other**		

Class II biological hood	Thermo Scientific	Cat# 51025411
CO2 incubator	Thermo Scientific	Cat# 3111
Centrifuge	Eppendorf	Cat# 5702000396
Inverted microscope (EVOS XL)	Thermo	Cat# AMF5000
Nucleofector 4D	Lonza	Cat# AAF-1002B
Li-Cor Odyssey 9120 Infrared Imaging System	LICOR	Cat# 22590
FACSCanto II	BD Biosciences	Cat# 338960
Confocal microscopy	Leica	TCS SP8
Freezers	Haier	HYCD-290
6-Well plates	Corning	Cat# 3516
24-Well plates	Corning	Cat# 3524
96-Well low-attachment V-bottom plates	Sumitomo Bakelite	Cat# MS-9096VZ

## Materials and equipment

### Aliquoting and plating matrigel

• At least 1 day before aliquoting, thaw a bottle of Growth Factor Reduced Matrigel (7–10 mg/mL, Corning) on ice at 4°C.
• Aliquot 2 mg of Matrigel into each pre-chilled 1.5-mL microcentrifuge tube using pre-chilled tips, all on ice. Immediately freeze the matrigel aliquots at −20°C or −80°C. It should be stored at −80°C and are stable for at least 1 year.

**CRITICAL:** Avoid multiple freeze-thaw cycles. Keep the Matrigel on ice during the entire aliquoting and plating process to prevent it from solidifying. Pipette tips and tubes should be also pre-chilled before use.

• Use 12 mL of ice-cold DMEM/F12 medium to thaw and resuspend each Matrigel aliquot (final concentration 0.16 mg/mL), which can be used to coat two 6-well plates.
• Mix the Matrigel well, add 1 mL of diluted Matrigel into each well of 6-well plates (250 μL into each well of 24-well plate), and ensure that the entire surface of plate well is covered.
• Incubate the plate at 37°C for 60 min, or at 4°C for 12 h.

**CRITICAL:** Matrigel should be removed from the freezer right before the experiment and should still be frozen when the DMEM/F12 medium is added.

***Note:*** The plates are incubated with Matrigel at 37°C for at least 30 min, but do not exceed 90 min. Minimize the amount of time that the coated plates are exposed to air. Drying can damage the Matrigel coating. Matrigel-Coated plates can be stored at 4°C for 2 weeks.

### 10 mM ROCK inhibitor Y-27632

• Dissolve ROCK inhibitor (Y-27632) in sterile DMSO or H_2_O to a final concentration of 10 mM (1,000×), and then aliquot and store it at −80°C. The solution is stable for at least 1 year.

### 1.1 M Taurine

• Dissolve 0.125 g Taurine in 10 mL sterile H_2_O and sterilize by filtration through a 0.22 μm filter. This makes a 0.1 M (1,000) stock that can be divided into 568 μL aliquots and store it at −20°C for 1 year.

### 5 mg/mL DNase I

• Reconstitute lyophilized DNase I (100 mg) in 20 mL sterile H_2_O. Aliquot and store at −20°C up to 6 months. Avoid freeze/thaw cycles.

### 5 mM Retinoic acid

• Dissolve 30 mg/mL retinoic acid in DMSO to obtain a master stock solution (100 mM), aliquot (vortexing may be needed) and store in light protected vials at −80°C. Master stock is reconstituted at 20× of the subsequent stock solution (5 mM) in DMSO and store at −20°C for up to 2 weeks.

***Note:*** Retinoic acid is more sensitive to light, heat, and air in solution. Protect it from light, heat, and air.

### 55 μg/mL recombinant human BMP4 (hBMP4)

• For a stock solution, reconstitute at 55 μg/mL in sterile 4 mM HCl containing at least 0.1% bovine serum albumin, aliquot, and store at −20°C for up to 3 months. hBMP4 can be stored at 4°C for up to 2 weeks once it is thawed.

***Note:*** Store the stock solution in a manual defrost freezer and avoid repeated freeze thaw cycles.

### hESC culture medium

• TeSR-E8 (Stem cell Technologies): complement 480 mL TeSR-E8 Basal Medium with 20 mL TeSR-E8 Supplement (25×). Store at 4°C for up to 2 weeks.

***Alternatives:*** Instead of TeSR-E8, other commercial hESC Culture Medium such as mTeSR-1 Medium, Essential 8 Medium (Thermo Fisher Scientific), and ncEpic Medium (Nuwacell) can be used.

### hESC dissociation solution

• 0.5 mM EDTA dissociation solution (1×): Add 500 μL of 0.5 M EDTA (pH 8.0) stock into 500 mL of DPBS (Ca^2+^/Mg^2+^-free). Add 0.9 g of sodium chloride (NaCl) and adjust the osmolarity to 340 mOsm, then filter with a 0.22 mm syringe filter and store at 4°C for up to 6 months.
• TrypLE Select solution Enzyme (1×): Supplement TrypLE Select Enzyme with 0.05 mg/mL DNase I and a final concentration of 20 μM Y-27632.

***Note:*** Once the DNase I and Y-27632 are added, the solution should be used within 1 week.

### hESC cryopreservation medium (2×)

• Add 2 mL of DMSO into 8 mL of TeSR-E8 to make 2× Cryopreservation Medium. Medium can be stored at 4°C for up to 1 week.

**Table:** Differentiation medium I

Reagent	Final concentration	Volume
IMDM	44% (v/v)	22 mL
Hams F12	44% (v/v)	22 mL
KSR	10% (v/v)	5 mL
GlutaMAX-I (100×)	1% (v/v)	0.5 mL
Monothioglycerol	450 μM	1.94 μL
Penicillin-Streptomycin (100×)	10,000 U/mL	0.5 mL
**Total**		**50 mL**

***Note:*** The dosage of Monothioglycerol must be particularly accurate, excess will affect cell aggregation. Store up to 2 weeks at 4°C. Protect from light.

**Table:** Differentiation medium II

Reagent	Final concentration	Volume
DMEM/F12	44% (v/v)	440 mL
FBS	10% (v/v)	50 mL
N2 supplement (100×)	1% (v/v)	5 mL
Penicillin-Streptomycin (100×)	10,000 U/mL	5 mL
5 mM stock Retinoic acid	0.5 μM	50 μL
0.1 M stock Taurine	100 μM	500 μL
**Total**		**500 mL**

***Note:*** After preparation, the differentiation medium should be stored at 4°C and used within 2 weeks.

**Table:** Cryopreservation pretreatment solution

Reagent	Final concentration	Volume
Differentiation Medium II	85% (v/v)	8.5 mL
ethylene glycol	5% (v/v)	0.5 mL
Sucrose	10% (w/v)	1 g
DMSO	10% (v/v)	1 mL
**Total**		**10 mL**

***Note:*** Filter the medium before adding ethylene glycol and DMSO. Store up to 2 weeks at 4°C. Protect from light.

**Table:** Organoid cryopreservation medium

Reagent	Final concentration	Volume
Differentiation Medium II	63.8% (v/v)	6.38 mL
Acetamide	1 M	0.59 g
propylene glycol	3 M	2.2 mL
DMSO	2 M	1.42 mL
**Total**		**10 mL**

***Note:*** Filter the medium with 0.22 μm filter before adding propylene glycol and DMSO. Store up to 2 weeks at 4°C. Protect from light.

## Step-by-step method details

### Thawing hESCs

**Timing: 30 min**

1. Prepare a Matrigel-coated 6-well plate (refer to Aliquoting and Plating Matrigel), and keep the hESC culture medium ready, which has been warmed to 22°C–25°C.
2. Remove the cryopreserved hESC vial from the liquid nitrogen storage tank, transfer to the 37°C water bath to thaw quickly.
3. When most the contents are thawed, slowly transfer it to 15-mL tube, and then add 5 mL of hESC culture medium in a drop wise manner. Gently shake the tube to mix the cells. Centrifuge at 200 × *g* for 5 min at 22°C–25°C.
4. Carefully aspirate and discard the supernatant, resuspend the cell pellet in 2 mL hESC culture medium supplemented with 10 μM Y-27632.
5. Completely aspirate Matrigel from one well of precoated 6-well plate, add the cell suspension into the well immediately, Place the plate into 37°C, 5% CO_2_ incubator, gently shake to evenly distribute the cells.

***Note:*** ROCK Inhibitor Y-27632 can markedly diminish dissociation-induced apoptosis of hESCs, enhance colony formation of dissociated hESCs after passaging.

**Figure 2:**
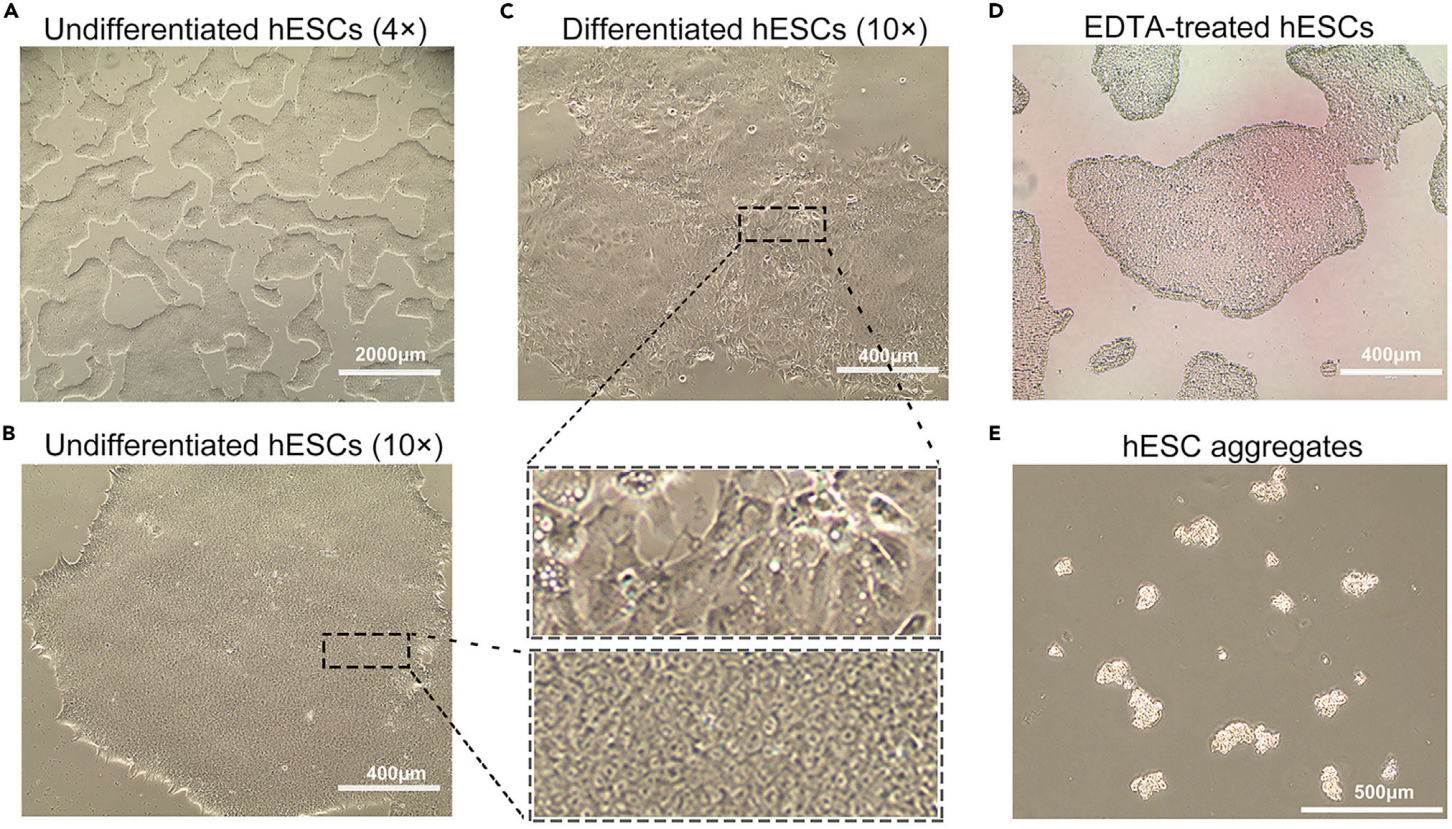
Representative photographs of hESCs grown on matrigel (A and B) Bright field images show cultured hESCs with undifferentiated state at 10× (A) and 4× (B) magnifications. The boxed region is magnified in the right. (A) Representative image shows an example of 60%–80% confluency of hESCs. (C) Representative image shows the morphology change of differentiated cell clones. The boxed region is magnified at the bottom. (D) hESC colony morphology after incubating with EDTA in a few minutes. (E) Optimal size of hESC aggregates during passaging with EDTA.

**CRITICAL:** Do not break apart the colonies too much by excess pipetting. See Figure 2E for optimal aggregate size.

### Feeding and passaging hESCs

**Timing: 60 min**

6. The next day after thawing hESCs, prewarm DPBS and hESC culture medium to 20°C–25°C, remove the spent medium with debris, wash the cells once with 1 mL prewarmed DPBS, and then add 2 mL fresh and prewarmed hESC culture medium without Y-27632 for continue feeding. Refresh culture medium daily until cells require passaging.

**CRITICAL:** Cell morphology is monitored under the inverted microscope. Healthy undifferentiated hESCs display round colony morphology with high cell density and clear boundary (Figures 2A and 2B), while the differentiated cells exhibit changed morphology (e.g., enlarged cells and cell-cell spacing, fibroblast-like morphology) (Figure 2C). Scrape off large differentiated colonies with a P1000 pipette tip by visual recognition. Small differentiated colonies will spontaneously disappear upon passaging.

7. Proceed to passage when the hESC colonies are becoming too large or reaching 60%–80% confluent (approx. 3–5 days).

***Note:*** For optimal results, cells should be approximately 60%–80% confluent after 3–5 days in culture (Figure 2B). If cells do not reach this confluency, adjust the timing to start the differentiation later.

8. Prewarm hESC culture medium, DPBS and EDTA Dissociation Buffer to 20°C–25°C. Prepare a Matrigel-coated 6-well plate (refer to Aliquoting and Plating Matrigel), aspirate the Matrigel from the well, and then add 2 mL of hESC culture medium supplemented with 10 μM Y-27632 per well.
9. Aspirate the spent medium from the hESCs to be passaged. Rinse the cells with 2 mL DPBS and EDTA solution sequentially, and then add 1 mL the EDTA solution to each well.
10. Incubate for 2–5 min at 22°C–25°C within the hood. Remove the EDTA solution carefully without disturbing the attached cell layer. Use 2 mL of hESC culture medium with 10 μM Y-27632 to wash the colonies off the plate and dissociate cells by gently pipetting up and down three times.

**CRITICAL:** The longer the hESCs incubated in the EDTA solution, the smaller the colonies that will result. Keep the movement of the plate to a minimum to avoid lifting the colonies completely off the plate during incubation (Figure 2D).

***Note:*** Do not break apart the colonies too much, avoid excessive pipetting as hESCs are very sensitive (Figure 2E).

11. Transfer the desired cell amount per well (the splitting ratio is about 1:6–1:10) into the readied Matrigel-coated plates.
12. Shake the plates back and forth and side to side to distribute the cells, leave it in the incubator for 12 h to allow maximum cell attachment.
13. On the next day, remove the culture medium from cells, rinse the cells with 1 mL prewarmed DPBS, and then add 2 mL of prewarmed hESC culture medium (without Y-27632) to each well in the 6-well plate.
14. On each following day, repeat step 13 to change culture medium, and monitor cells daily.

***Note:*** Long time culture (exceeding 48 h) in Y-27632 conditioned culture medium will cause significant irreversible changes in cell morphology, thus it may affect the cell state of hESCs. The conditioned medium should be replaced by hESC culture medium (without Y-27632) the next day after thawing or passaging.

**CRITICAL:** Passages of stem cell always have impacts on the subsequent applications, we recommend using hESCs between passages 30 and 60. For subsequent applications, it is recommended to use hESCs that have been passaged at least twice after thawing to ensure the cells have returned to normal status.

### Generation of biallelic *RB1*-mutated (*RB1*^Mut/Mut^) hESC lines using CRISPR/Cas9-mediated genome editing

**Timing: 8–12 weeks**

15. When hESC colonies are reaching 60%–80% confluent (Figure 2B), aspirate culture medium, rinse the cells with 1 mL prewarmed DPBS and TrypLE Select solution (refer to hESC dissociation solution) sequentially, and then incubate with another 1 mL TrypLE Select solution for 3–5 min at 37°C.

***Optional:*** Pretreatment with 10 μM Y-27632 in hESC culture medium for 2 h before single-cell plating can effectively enhance the survival rate of hESCs (Liu et al., 2016).

***Alternatives:*** Other commercial reagents (i.e., ACCUTASE) for single cell dissociation can be used as a substitute.

16. Using P1000 pipette tip, gently pipette up and down the TrypLE Select solution in the well three times to make a single-cell suspension.

**CRITICAL:** Avoid excessive pipetting as hESCs are very sensitive. Monitor dissociation under a bright-field microscope to ensure that cells are dissociated into single cells. If some cell lines appear to be more difficult to dissociate, the dissociation time can be extended to 10 min.

17. Transfer the single-cell suspension to a 15-mL centrifuge tube containing 5 mL of hESC culture medium.
18. Cell density is counted using a Handheld Automated Cell Counter (Millipore). Meanwhile, centrifuge the cell suspension at 200 × *g* for 5 min.
19. Remove the supernatant, and approximately 2 × 10^6^ cells are re-suspended with nucleofector solution prepared according to the manufacturer’s protocol by mixing 82 μL P3 primary cell solution and 18 μL supplement 1 (Lonza), mixed with 5 μg of plasmid cocktail, including 2.5 μg of guide-carrying plasmids and 2.5 μg of *RB1* targeting vector, and transferred into a nucleofection cuvette (Lonza).
20. Cells in the nucleofection cuvette are electroporated under the program CA-137 using Nucleofector 4D (Lonza).

***Note:*** Avoid trapping bubbles when transferring, as it may impact the efficiency of electroporation.

***Alternatives:*** Other programs or Nucleofector equipment for hESCs electroporation can be optimized or tested to achieve the best transfection efficiency.

**Figure 3:**
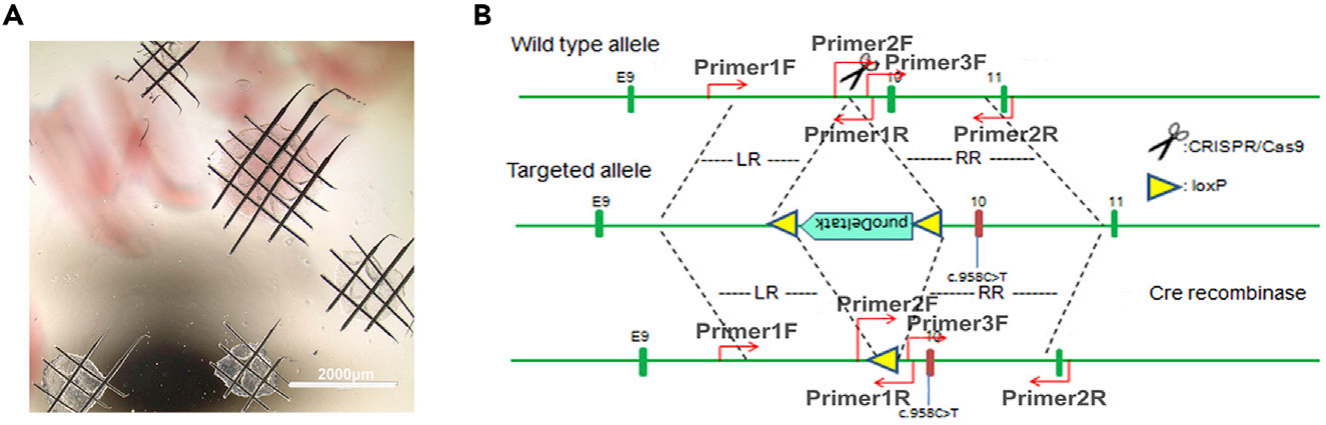
Generation and identify of Biallelic *RB1*-mutated (*RB1*^Mut/Mut^) hESC lines (A) Bright field image shows the survived single-clones after resistance selection can be picked by mechanically cross-hatch the colony. (B) The location of the primers used to identify the recombination.

21. Following nucleofection, gently transfer the cells into Matrigel-coated plates containing hESC culture medium supplemented with 10 μM Y-27632, and cultured in a 37°C, 5% CO_2_ incubator.
22. 48 h after electroporation, treat cells with 2 μg/mL puromycin (Gene Operation) for about 7 days. After puromycin selection, the surviving clones will appear and should be ready for picking, expanding and for further genotyping.
23. For picking clones, prepare a Matrigel-coated 24-well plate (refer to Aliquoting and Plating Matrigel). After coating, replace Matrigel with 0.5 mL of hESC culture medium supplemented with 10 μM Y-27632 per well.
24. Find cell colonies under the microscope. By using a smaller-gauge needle, cross-hatch the colony so that it will come off the plate in smaller pieces (Figure 3A). Use a P1000 pipette tip to push the colony off the plate and suck it into a pipette tip. Transfer the colony pieces into one well of the 24-well plate.

***Note:*** Pick the colonies under a normal inverted microscope. The microscope can be placed inside a Class II biological hood to allow for a sterile field while picking colonies.

25. Repeat steps 23 and 24 with other colonies. Place the plate into 37°C, 5% CO_2_ incubator, and gently shake to evenly distribute the cells.
26. Next, the colonies are expanded as described in steps 6–14.
27. Identification of introduced mutations in positive *RB1*^Mut/Mut^ clones (named as *RB1*^Mut/Mut^ hESCs).
  a. Extract genomic DNA from individual colonies using DNeasy Blood & Tissue Kit according to manufacturer's instructions.
  b. Detect the HR (Homologous recombination) and non-HR allele by PCR using the following primers (Figure 3B): Primer1F (5′-GTGCTAGTGGGAGAGGCTGACAAAG-3′) / Primer1R (5′-GCCAATGCTACAAAACAGTACCTAA-3′), Primer2F (5′-CACGCCTGTAATTCTAGTACTTTGG-3′) / Primer2R (5′- GAAACGTGAACAAATCTGAAACACT -3′) or Primer3F (5′- TGGGATGTTTGGAAAATCTTGGCAGT -3′) / Primer2R,
  c. Examine the PCR fragments by Sanger sequencing.
  d. Identify potential mutations of *RB1* gene by comparing the colony sequence with WT sequence.
28. Routinely maintenance of *RB1*^Mut/Mut^ hESCs is same with WT hESCs, as described in steps 1–14.

***Optional:*** Ideally, resistance gene (puro) in *RB1*-mutated hESCs can be further removed using the Cre/LoxP system.

***Alternatives:*** Here, we describe the generation of human Rb organoids from biallelic *RB1*-mutated (*RB1*^Mut/Mut^) hESCs. hESCs with a biallelic *RB1* knockout (*RB1*^−/−^) (Avior et al., 2017) can also be used to establish the human Rb organoids, as described in our publication (Liu et al., 2020).

**CRITICAL:** Only biallelic *RB1*-mutated or knockout hESCs with the loss of RB protein function can be used effectively to generate Rb organoids.

### Characterization of *RB1*^Mut/Mut^ hESC lines

**Timing: 5–7 days**

With the knockin of nonsense mutation p.R320X, the generated *RB1*^Mut/Mut^ hESC lines should absent the expression of RB protein (pRB), and sustain the primordial state without changing pluripotency and genetic integrity.

29. Perform western blotting with anti-Rb antibody at 1:1,000 dilutions to analyze the expression of RB protein (pRB) in *RB1*^Mut/Mut^ clones.
30. Flow cytometry analysis for pluripotency marker genes (i.e., Oct3/4 or Sox2) in *RB1*^Mut/Mut^ clones.
  a. Use prewarmed TrypLE Select solution to carefully dissociate the clones into single-cell suspension as described in steps 15–18.
  b. Repeat PBS washes twice and resuspend cells in media.
  c. Stain the cells with Alexa Fluor 488/647-conjugated antibodies (1:50) in PEB (PBS containing 0.5% BSA and 2 mM EDTA) buffer for 30 min at 4°C.
  d. The cells are filtered through 100 μm nylon mesh and then analyzed for fluorescence by FACSCanto II (BD Biosciences).
31. Karyotype analysis of *RB1*^Mut/Mut^ clones.
  a. Aspirate the culture medium, and add 1 mL of DPBS to wash out the remnants of the medium.
  b. Aspirate the DPBS, and treat the cells with 0.1 μg/mL colcemid at 37°C for 2 h.
  c. Use prewarmed TrypLE Select solution to carefully dissociate the clones into single-cell suspension as described in steps 15–18.
  d. Resuspend and incubate the cells in 0.075 M potassium chloride for 15 min at 37°C.
  e. Fix the cells with 3:1 methanol:acetic acid, and then drop onto slides to spread the chromosomes.
  f. The chromosomes can be visualized by Giemsa (Servicebio) staining.

### Single-cell seeding, embryoid body (EB) formation, and neuroepithelium induction

**Timing: 18 days**

32. **On day 0**, check the cell confluency, 60%–80% is the best for starting the differentiation (Figure 2B). Use prewarmed TrypLE Select solution to carefully dissociate *RB1*^Mut/Mut^ hESCs into single-cell suspension as described in steps 15–18.
33. Count cell density, and plate at a density of 12,000 live cells per well in 96-well low-attachment V-bottom plates with 100 μL differentiation medium I supplemented with 20 μM Y-27632.

***Note:*** The plating density affects cell viability and differentiation efficiency. We recommend using the cell density at 12,000 live cells/well. Approximately 144×10^4^ cells needed per 96-well low-attachment V-bottom plate, and re-suspended with 12 mL differentiation medium I supplemented with 20 μM Y-27632. Plate 100 μL cell suspension into each well of a 96-well low-attachment V-bottom plate.

**Figure 4:**
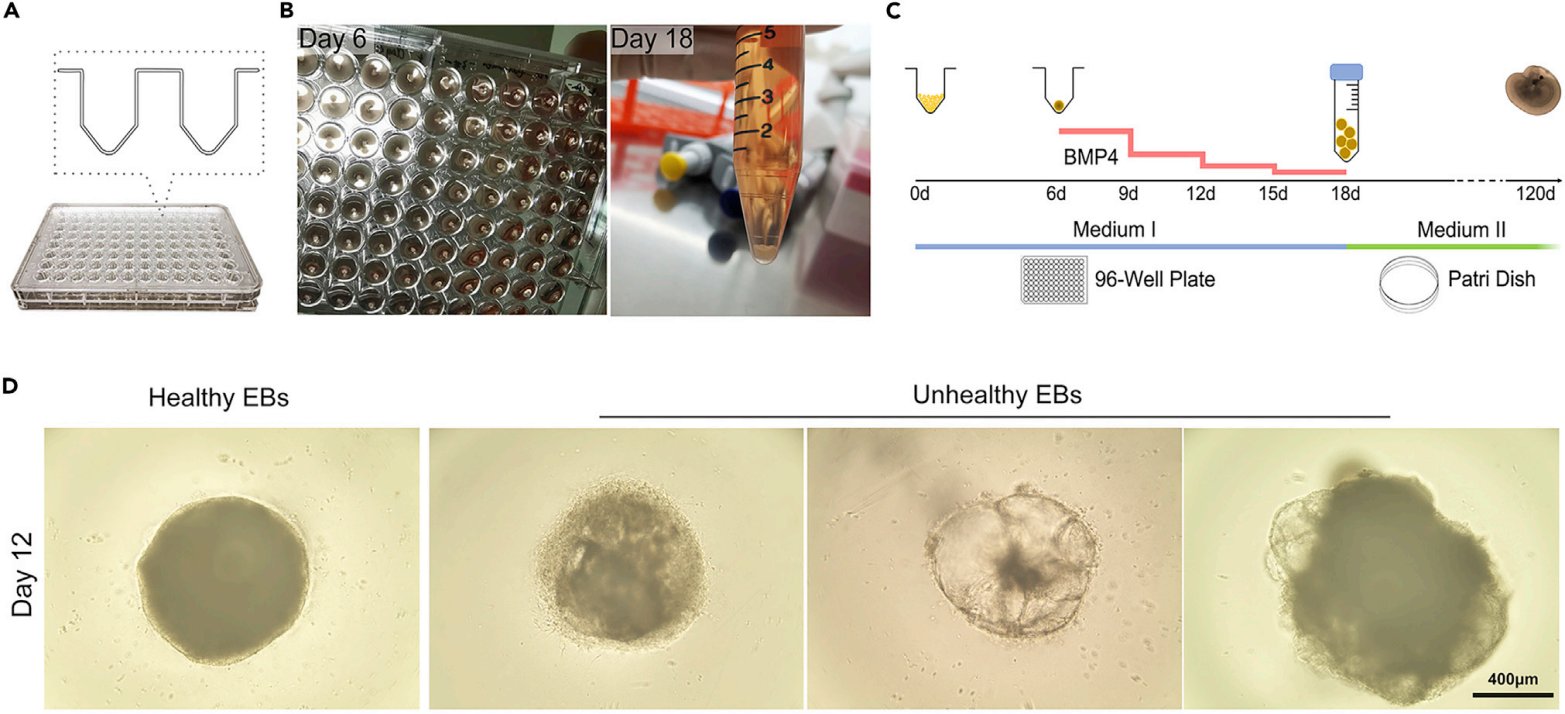
Single-cell seeding, EB formation, and neuroepithelium induction (A) 96-well low-attachment V-bottom plates used for rapid cell reaggregation. (B) Aggregates can be visible with the naked eye from day 6. When transfer the organoids from each well into a 15-mL tube at day 18, the organoids should gather at the bottom of the tube after few minutes of standing. (C) Stepwise induction strategy of Rb organoids. Timed hBMP4 treatment is critical for differentiation induction. (D) Representative images of the healthy and unhealthy EBs at differentiation day 12.

**CRITICAL:** 96-well low-attachment V-bottom plates are needed for rapid cell reaggregation (Figure 4A). Y-27632 can also significantly increase cell reaggregation.

34. Put the plates on a spiral mixer device, and shake for 10 min at 60–80 rpm to allow cells to gather at the bottom of the well.
35. Place the plates into 37°C, 5% CO_2_ incubator.
36. **On day 6**, take out the plate and slightly tilt it, use an 8-channel P100 pipette to carefully aspirate the medium from each well, leaving EB-like aggregates at the bottom of wells. Aggregates in each well can be visible with the naked eye (Figure 4B).
37. Quickly add 100 μL fresh differentiation medium I containing 55 ng/mL hBMP4 to each well to re-suspend the aggregates, and incubate at 37°C, 5% CO_2_ for 3 days.
38. **On day 9**, carefully remove 50 μL medium from each well, change equal volume of fresh differentiation medium I (without hBMP4) back to each well for 3 additional days (Figure 4C).
39. **On day 12**/**day 15**, perform routine half medium change as described in step 38.

***Note:*** Optimal EBs should have a round and smooth morphology, the anterior neuroepithelium develops on the outer surface and is quite optically translucent (Figure 4D). Suboptimal EBs usually exhibit uneven surface with dead or unhealthy cells attached, fails to form neuroepithelium, and will eventually form cystic organoids (Figure 4D).

**CRITICAL:** Timed hBMP4 treatment is critical for differentiation induction. A volume of 55 ng/mL human BMP4 is added to the culture on day 6, and its concentration is semi-reduced with a half medium change every 3 days (Figure 4C).

### Early stage of retinal differentiation

**Timing: 6 weeks**

40. On day 18, take out the plate and gently tap from both sides to make the aggregates (organoids) free-floating. Organoids can be visible with the naked eye (Figure 4B). Using a wide-mouth P1000 pipette tip (simply cut the tip with scissors), transfer the organoids from each well into a 15-mL conical tube.

**CRITICAL:** To avoid the damage of aggregates, use wide-mouth pipette tips to transfer aggregates. Gentle operation is also required for the transfer process.

41. Let the organoids to settle at the bottom of the tube (approx. 1–2 min) (Figure 4B) and carefully remove the supernatant from the top.
42. Add 5 mL differentiation medium II to wash the organoids, let the organoids settle to the bottom again and then remove excess differentiation medium II.
43. Carefully resuspend the organoids with 10 mL differentiation medium II and transfer into a low attachment 9-cm Petri dish using a 10-mL pipette.
44. Manually trisect the organoids using a V-Lance Knife (Alcon Surgical) under the inverted microscope in a Class II biological hood (Methods video S1). Add 10 mL of additional differentiation medium II to the Petri dish.

**CRITICAL:** Turn off the light of the hood to avoid isomerization of retinoic acid in differentiation medium II.

45. Gently shake the dish and incubate at 37°C, 5% CO_2_ /40% O_2_ for continue induction.
46. Refresh the differentiation medium II every 7 days (Methods video S2).
  a. For media refreshing, rotate the dish to gather the organoids in the center.
  b. Carefully aspirating the medium from the surrounding using vacuum aspiration system.
  c. Aspirate as much media as possible without disturbing organoids.
  d. Replace with 20 mL fresh differentiation medium II.

Methods video S1. Manually trisect the organoids using a V-Lance Knife under the inverted microscope in a Class II biological hood, step 44

47. Gently swirl the Petri dish every day to avoid the organoids adhere to the bottom.
48. On day 20, take out the dish, remove the badly differentiated organoids using a 10-mL pipette, and separate the fused organoids using a V-Lance Knife under the inverted microscope. Place the dish into incubator for further culture.

**Figure 5:**
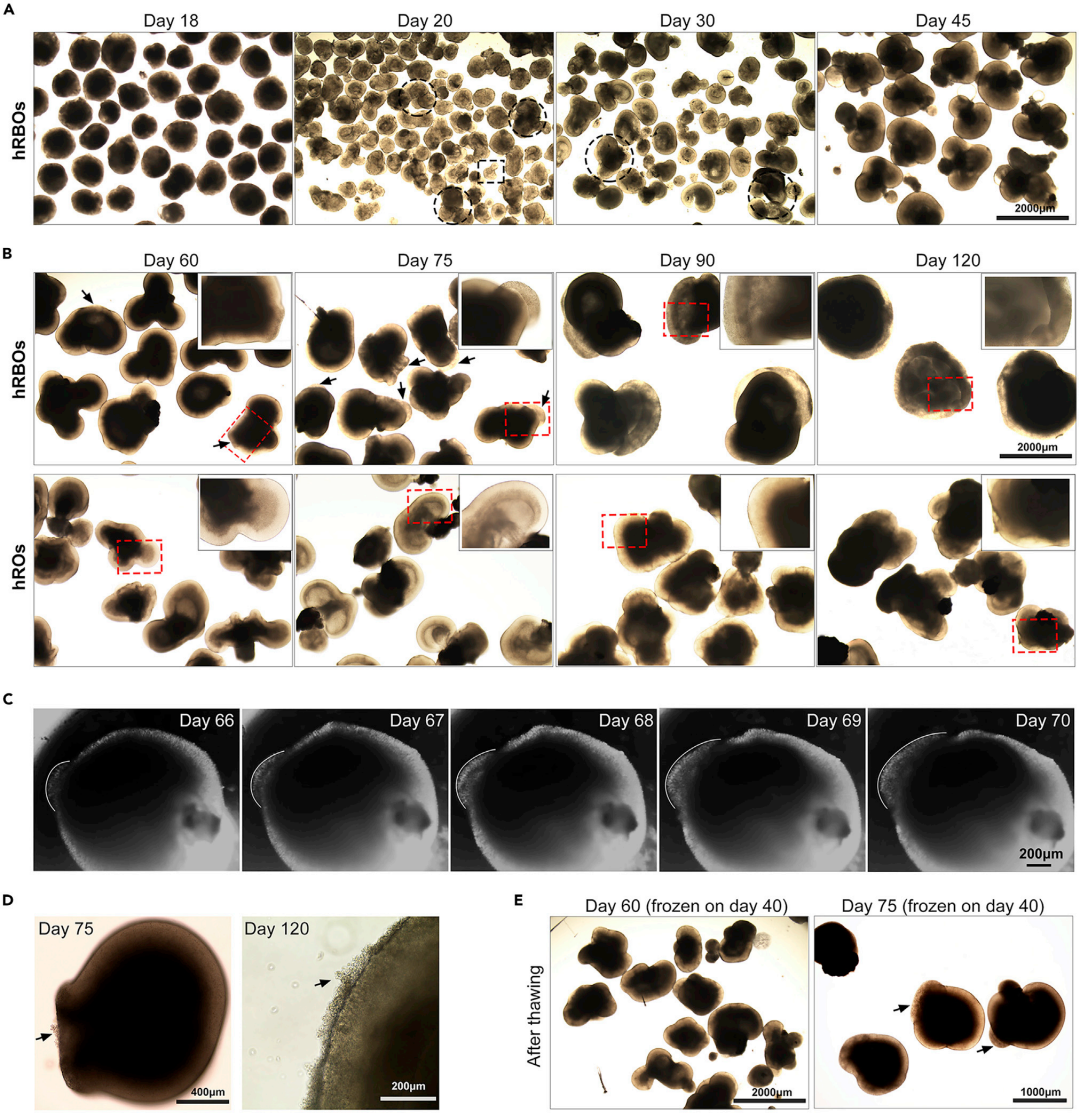
Retinal differentiation and tumorigenesis of human Rb organoids (A) Representative images show the morphologic features of hRBOs at early stage of retinal differentiation (Day 18, 20, 30 and 45). Dashed lines denote the fused organoids (circular) or the adhered organoids (square). (B) Representative images show the morphologic features of hRBOs at late stage of tumor induction (Day 60, 75, 90 and 120). The boxed region is magnified in the insets. Arrows indicate tumor-like “primary foci” structures. Wild-type hESC-derived human retinal organoids (hROs) were used as the control (Bottom row). (C) Representative images show the rapid expansion of tumor-like structures in developing hRBOs (Days 66–70). (D) Representative bright-field image of 75- and 120-day-old hRBOs. Arrows indicate excessive outgrowth of Rb cells, subsequently leading to detachment from the organoids. Scale bar, 400 μm. (E) A typical morphology of NR and tumor-like “primary foci” structures in hRBOs can be observed after thawing.

***Note:*** The state and morphology of organoids can monitor in real time using an inverted microscope (Figures 5A and 5B). Organoids in poor condition tend to adhere to the bottom of Petri dish (Figure 5A), pipette the adhered organoids with the medium to resuspend it, or remove it using a 10-mL pipette.

Methods video S2. Media refreshing for hRBOs in culture plate, step 46 Rotate the dish to gather the organoids in the center, carefully aspirating the medium from the surrounding using vacuum aspiration system, aspirate as much media as possible without disturbing organoids, replace with fresh medium finally.

***Alternatives:*** When organoids are culture in suspension in a Petri dish, half medium can be changed every 5 days.

49. On day 30, repeat step 48 to remove the badly differentiated organoids, separate the fused organoids under the inverted microscope (Figure 5A).
50. Using a 10-mL pipette, gently distribute the organoids into multiple dishes, with no more than 30 organoids in each dish. Return the dishes to 37°C, 5% CO_2_ /40% O_2_ incubator, change the medium every 7 days as described in step 46.

**CRITICAL:** Do not put too many organoids in a single plate, which is not conducive to long-term culture.

***Note:*** There was no observable difference between Rb and retinal organoids before differentiation day 45 (Figures 5A and 5B).

### Tumorigenesis and long-term culture of human Rb organoids

**Timing: 5 weeks**

51. On day 60, tumor-like “primary foci”, with the obvious uneven density inside and ill-defined edge, are visible in a minority of retinal organoids (named as human Rb organoids) under the microscope (Figure 5B). It will expand rapidly from the masses thereafter (Figures 5B and 5C).
52. Perform media change routinely every 5–7 days as described in step 46.
53. On day 75, obvious tumor-like “primary foci” structures can be seen in most organoids (Figure 5B).
54. Using a 10-mL pipette, gently distribute the organoids into multiple dishes, with no more than 15 organoids in each dish, refreshing the medium every 5 days as described in step 46.

**CRITICAL:** The tumor structures are relatively loose and easy to detach from the organoids. Be careful when changing the medium or collecting samples to avoid damage of Rb organoids (Figure 5B).

55. After day 90, the tumor-like “primary foci” structures will wrap around the entire mass (Figure 5B), and excessively proliferating Rb cells can migrate into the medium in suspension conditions (Figure 5D).
56. Gently trisect the larger organoids using a V-Lance Knife under the inverted microscope for long-term culture.

***Note:*** Avoid organoids from growing too large, which will cause nutrient deficiency in the cells inside the organoids.

### Characterization and tumor growth monitoring of human Rb organoids

**Timing: 5–7 days**

For the characterization of human Rb Organoids, they can be cryopreserved, sectioned, and immunocytochemically stained for the expression of proliferative or Rb marker, such as Ki67, SYK, DEK and p16^INK4a^.

57. After day 75, select the organoids with obvious tumor-like “primary foci”, slightly transfer into a 2-mL using a 10-mL pipette.
58. Carefully aspirate the supernatant, wash the organoids three times with 1 mL DPBS, then add 0.5 mL 4% paraformaldehyde (PFA) solution to fix them at 37°C for 1 h. Wash the organoids three times with PBS.
59. Stain the organoids with Ponceau S solution at 37°C for 5 min, and then wash them with NEG-50 Frozen Section Medium.
60. Carefully embed the organoids into a disposable embedding mold containing NEG-50 Frozen Section Medium.
61. Quickly place the molds into −80°C refrigerator for 20–30 min to freeze the organoids, and then cryosection at a thickness of 12–16 μm on slides using a cryostat.

**Pause point:** Once fixed and embedded, organoids can be stored at −80°C for more than 12 months. After cryosection, the sections can subject to immunocytochemistry or storage at −80°C for up to 12 months

62. For immunocytochemistry, wash the sections three times for 10 min with PBS, block and permeabilize the sections in 4% BSA with 0.5% Triton X-100 for 1 h at 22°C–25°C.
63. Dilute the primary antibody in PBS with 1% BSA with 0.5% Triton X-100.
64. Aspirate the spent blocking buffer of the sections, and incubate with appropriate dilutions (1:100–1:400) of primary antibody at 4°C for 12 h.

***Note:*** Different primary antibodies may require specific immunostaining conditions, please refer to the manufacturer’s instructions.

65. Dilute the secondary antibody (1:400) in PBS with 1% BSA with 0.5% Triton X-100.
66. Aspirate the primary antibody dilution, wash three times for 10 min with PBS, add 100–200 μL secondary antibody dilution, and incubate for 1 h in the dark at 22°C–25°C.
67. Aspirate the secondary antibody dilution and wash once with PBS. Add 300 nM DAPI nuclear stain solution and incubate for 5-10 min in the dark at 22°C–25°C.
68. Remove the solution and wash the sections three times with PBS.
69. Using absorbent paper absorb moisture around the sections, add 10 μL antifade mounting medium, and gently cover the cover slips. Seal the edges of the coverslip using transparent nail varnish and dry for 3 min. Store the slides at 4°C in the dark.
70. The stained sections can be visualized using confocal microscopy.

### Cryopreservation of human Rb organoids

**Timing: 1.5 h**

Organoids are typically cryopreserved intact at the relatively early stage (day 30–50) and can be used for further applications after thawing.

71. **During days 30–50**, take out the dishes, carefully collect the organoids using a 10-mL pipette and transfer into a 15-mL tube containing 1 mL differentiation medium II.
72. Place the tube on ice for 10 min.
73. Remove the supernatant from the top, replace with 1 mL pre-chilled cryopreservation pretreatment solution, and incubate on ice for 10–15 min.
74. Remove the Pretreatment Solution, add pre-chilled organoid cryopreservation medium to resuspend the organoids.
75. Transfer 200 μL of the cryopreservation medium with 15–20 organoids to a labeled 1.5-mL cryovial.
76. Directly frozen the vials in liquid nitrogen for storage.

**CRITICAL:** Do not use narrow-mouth pipette tip to collect or transfer the organoids, which will seriously affect the survival efficiency of frozen organoids.

***Note:*** Human Rb organoids can be cryopreserved for at least one year using this protocol.

77. For organoid thawing, take out the stock vials from liquid nitrogen, add 1 mL prewarmed (20°C–25°C) differentiation medium II to thaw the frozen human Rb organoids quickly.
78. Remove the supernatant, and then wash twice with differentiation medium II.
79. Transfer the organoids to a new Petri dish using a 10-mL pipette, add 15 mL of differentiation medium II for post-maintenance culture.

***Note:*** Five days after thawing, neural retina-like structures are clearly visible in the organoids that are alive and in good condition (Figure 5E). In our experiences, the survival rate of Rb organoids after thawing is ranges between 60%–80%.

## Expected outcomes

As a genetically related malignancy, Rb is caused by *RB1* mutations. This protocol describes an efficient method to generate an in-dish human Rb organoid system from the genetically engineered embryonic stem cells with a biallelic *RB1*-mutation (*RB1*^Mut/Mut^).

The key to establish this Rb organoid system is to generate gene-edited human embryonic stem cells (hESCs) with a biallelic *RB1*-mutation (*RB1*^Mut/Mut^) at the first stage. In our experience, only biallelic *RB1*-mutated hESCs with the loss of RB protein function can be used effectively to generate human Rb organoids. Using this protocol, the efficiency of homozygous *RB1*-mutation knock in clones is about 10%–15% (5 out of 40 in puro-resistant clones). It takes 2–3 months for generation of these biallelic *RB1*-mutated hESCs. We have identified that healthy *RB1*^Mut/Mut^ hESC lines sustains the primordial state without changing pluripotency, genetic integrity as well as the cell cycle of the hESC lines (Liu et al., 2020).

*RB1*^Mut/Mut^ hESC lines can further differentiate into human Rb organoids in a stepwise manner. Like normal retinal organoid derived from WT hESCs (Kuwahara et al., 2015; Jin et al., 2019; Pan et al., 2020), the morphogenetic and molecular properties of *RB1*^Mut/Mut^ hESC-derived Rb organoids recapitulate the developing human retina normally before the differentiation day 60, i.e., multilayered neural retina (NR) containing all retinal cell types (Liu et al., 2020). Remarkably, tumor-like “primary foci” were clearly visible in Rb organoids at day 60–75(Figure 5B). These tumor-like structures exhibited an obvious uneven density inside, ill-defined edge and larger size (Figures 5B–5E). And these tumor-like foci expanded rapidly from the masses thereafter (Figure 5C). At this early stage (day 60–75, referring to Rb organoids at the onset of tumorigenesis), the derived organoids can be analyzed for Rb characteristics (e.g., molecular signatures, histological features, tumorigenicity *in vivo*) and applied in further experiments (Liu et al., 2020). After ages of day 90, the derived Rb organoids with significant tumorigenesis were relatively “mature” and homogeneous (Figure 5B). And the tumor-like “primary foci” structures wrapped around the entire organoids, and excessively proliferating Rb cells can migrate into the medium in suspension conditions (Figures 5B and 5D).

As described in our original paper (Liu et al., 2020), these Rb organoids exhibit properties highly consistent with Rb tumorigenesis, transcriptome, and genome-wide methylation.

This organoid system offers an innovative, convenient, and yet elegant model that can be used efficiently and effectively for dissecting the origination of tumor cells and mechanisms of Rb tumorigenesis as well as for screening of novel therapies in terms of efficacy and safety.

## Limitations

One limitation of this protocol is that differentiation efficiency varies substantially by the state (including the pluripotency, self-renewal ability and differentiation capability) of the cultured hESCs and their derivatives, which might be attributed to genetic background, frozen batches, passages, and culture conditions. In addition, given the loss of *RB1* function in the genetically engineered hESCs at the beginning of differentiation, all cells in the Rb organoids derived from these hESCs will lose *RB1* function, which will also lead to significant decrease of normal retinal cells in this model system, which may be not suitable for studying tumor microenvironment, invasion or metastasis.

## Troubleshooting

### Problem 1

Failure in establishing *RB1*^Mut/Mut^ hESCs with the point mutation of c.958C>T; p.R320X.

### Potential solution

Be sure that the constructed guide-carrying and targeting vector are effective. Before transfecting hESCs, we recommend verifying their effectiveness on 293T cells.

In addition, hESCs are difficult to transfect (Liu et al., 2016), and efficient transfection is the key to successfully obtaining the gene-edited hESCs, so the transfection efficiency needs to be improved as much as possible. We routinely get 50%–60% of transfection efficiency. Due to the difference in cell lines and experimental conditions, the recommended program of nucleofection in this protocol can be appropriately adjusted to achieve efficient transfection.

As an alternative, hESCs with a biallelic *RB1* knockout (*RB1*^−/−^) can be generated and further used to establish the human Rb organoids, as we demonstrated in our recent publication (Liu et al., 2020).

### Problem 2

Low efficiency to form embryoid bodies.

### Potential solution

Adjust cell state of hESCs and their derivatives. The efficiency of EB formation can be seriously impacted by the state of the cultured hESCs and their derivatives. Adjust the cell state before single-cell seeding by optimizing the culture conditions for expansion, transduction, passage, cryopreservation, and recovery, or changing the culture medium. We achieve best results using hESC culture medium, TeSR-E8 (Stem cell Technologies).

Optimize Single-cell Seeding. ROCK inhibitor (Y-27632) is described as required for single-cell passaging (10 μM) and seeding (20 μM) in this protocol. ROCK inhibitor can significantly improve the survival rate of single cells and greatly increase cell reaggregation. In addition, 96-well low-attachment V-bottom plates are critical for rapid cell reaggregation. PrimeSurface 96-well low-attachment V-bottom plates (Sumitomo Bakelite) do work well in this protocol and are recommended.

Lastly, plating density should be accurately controlled, which also affect the formation of EBs and subsequent differentiation (Jin and Takahashi, 2012). A low number of hESCs per well leads to small embryoid bodies with insufficient differentiation capacity, while an excessive number of cells per well results in large embryoid bodies with apoptotic cells. The recommended planting density (12,000 live cells/well) is suitable for most of the stem cell lines (Deng et al., 2018; Pan et al., 2020). For different cell lines, optimization may be needed to obtain optimal plating density if the efficiency of EB formation is still low after several attempts.

### Problem 3

No tumorigenesis happens in the organoids.

### Potential solution

Tumorigenesis in the derived Rb organoids usually occurs from day 60 to 75. A possible explanation for the lack of visible tumor might be RB1 heterozygous mutation or mutation loss in genetically engineered hESCs or its derived organoids. Only biallelic *RB1*-mutated or knockout hESCs with the loss of RB protein function can be used effectively to generate Rb tumor. Confirm the expression of RB protein (pRB) is absent in *RB1*^Mut/Mut^ hESCs and its derived organoids.

Another possible reason is that retinal organoids induction fails in the early stage of differentiation. Make sure the cellular compositions and morphogenetic properties of the early-stage organoids can recapitulate the developing human retina. If not, modify differentiation conditions or avoid damage to organoid integrity during induction to generate the healthy early-stage organoids containing all major retinal cell types, especially cone precursors, which are the cell-of-origin of Rb (Liu et al., 2020; Singh et al., 2018). Spontaneously degenerating retinal organoids sometimes show morphology similar to Rb organoids, but they have essential differences. For example, due to the abnormal proliferation of tumor cells and tumor foci, Rb organoids have obvious proliferation and expansion trends, while the degenerating organoids lacks this tendency. This results in Rb organoids being generally larger than degenerating organoids. Of course, by identifying the expression of tumor markers, it is easy to distinguish these two types of organoids (Liu et al., 2020).

## Resource availability

### Lead contact

Further information and requests for resources and reagents should be directed to and will be fulfilled by the lead contact, Zi-Bing Jin (jinzibing@foxmail.com).

### Materials availability

This study did not generate new unique reagents.

### Data and code availability

This study did not generate any unique datasets or code.
